# The effect of a centralization procedure for extruded lateral meniscus on load distribution in porcine knee joints at different flexion angles

**DOI:** 10.1186/s12891-020-03197-2

**Published:** 2020-04-03

**Authors:** Rei Kubota, Hideyuki Koga, Nobutake Ozeki, Junpei Matsuda, Yuji Kohno, Mitsuru Mizuno, Hisako Katano, Ichiro Sekiya

**Affiliations:** 1 Center for Stem Cell and Regenerative Medicine, Tokyo Medical and Dental University, 1-5-45 Yushima, Bunkyo-ku, Tokyo, 113-8510 Japan; 2 Department of Joint Surgery and Sports Medicine, Graduate School, Tokyo Medical and Dental University, Bunkyo City, Japan

**Keywords:** Meniscus, Meniscal extrusion, Centralization, Load distribution analyses

## Abstract

**Background:**

Meniscal extrusion results in loss of the ability to resist hoop strain and biomechanical overload on the joint articular surface. A centralization technique has been developed to overcome these problems. In this study, we analyzed the biomechanics of the extruded and centralized lateral meniscus (LM) in porcine knee joints at different flexion angles.

**Methods:**

Porcine knee joints (*n* = 8) were set in the universal tester and each knee was tested under the following states: 1) intact; 2) extrusion—meniscal extrusion was created by resecting the posterior root of the LM and posterior synovial capsule; and 3) centralization—centralization was performed by two anchors inserted in the lateral tibial plateau. Deviation distance of the meniscus, contact pressure, and contact area in the anterior LM, middle LM, posterior LM, and the contact pressure of the tibial cartilage were evaluated with an axial compressive force of 200 N at knee flexion angles of 30°, 45°, 60°, and 90°.

**Results:**

The deviation distance of LM significantly increased in extrusion but was restored to the intact status after centralization at all angles. Both the contact pressure and area significantly decreased in extrusion and were restored after centralization close to the intact status in the anterior and middle LM; in the posterior LM, however, decreased contact pressure and area were not restored after centralization. The contact pressure of the tibial cartilage increased significantly in extrusion but decreased close to the intact status after centralization.

**Conclusions:**

This centralization procedure could reduce extrusion of the LM and restore the load-distributing function of the anterior-middle LM. However, the procedure itself could not restore hoop function in cases where the defect lies in the posterior LM.

## Background

Meniscal extrusion induces dysfunction of load distribution, one of the most important functions of the meniscus [1–3]. It is caused by the disruption of the meniscus hoop function and is often observed after meniscectomy [1, 4], meniscus root tears [5], and with aging [6–8]. Meniscal extrusion initiates osteoarthritis (OA) and accompanies its progression [9–11]. Restoring the lost function caused by meniscus extrusion can delay OA progression [12].

A centralization technique has been developed to reduce meniscal extrusion; the capsule attached to the meniscus is sutured to the edge of the tibial plateau using suture anchors [13]. Arthroscopic centralization of the extruded lateral meniscus (LM) improved clinical outcomes at two-year follow-up [14]. It also increased the radiographic lateral joint space width on standing at the 45° flexion view at 3 months; this was maintained for 2 years [14].

Biomechanical studies examining the effects of centralization are still limited, although some papers were recently published [15–17]. We already reported the biomechanical analysis of the centralization procedure for extruded LM with posterior root deficiency in a porcine model. Although this study showed that the centralization procedure restored the load distribution to a value closer to that of the normal knee joint, the experiment was performed only at 45° of knee flexion [15] and the effects of centralization in the more extended and more flexed knee position remained unknown. The purpose of the current study was, therefore, to analyze the effects of the centralization procedure in porcine knee joints at different flexion angles (as well as at 45°) in order to further clarify the biomechanical properties of the centralization procedure.

## Methods

### Porcine knee joints

We used porcine knee joints (Tokyo Shibaura Zouki, Tokyo, Japan), which were fresh-frozen and only right-side. We excluded knees with damaged meniscus or cartilage. We analyzed only the lateral compartment in eight right knees.

### Experimental setup

**Fig. 1:**
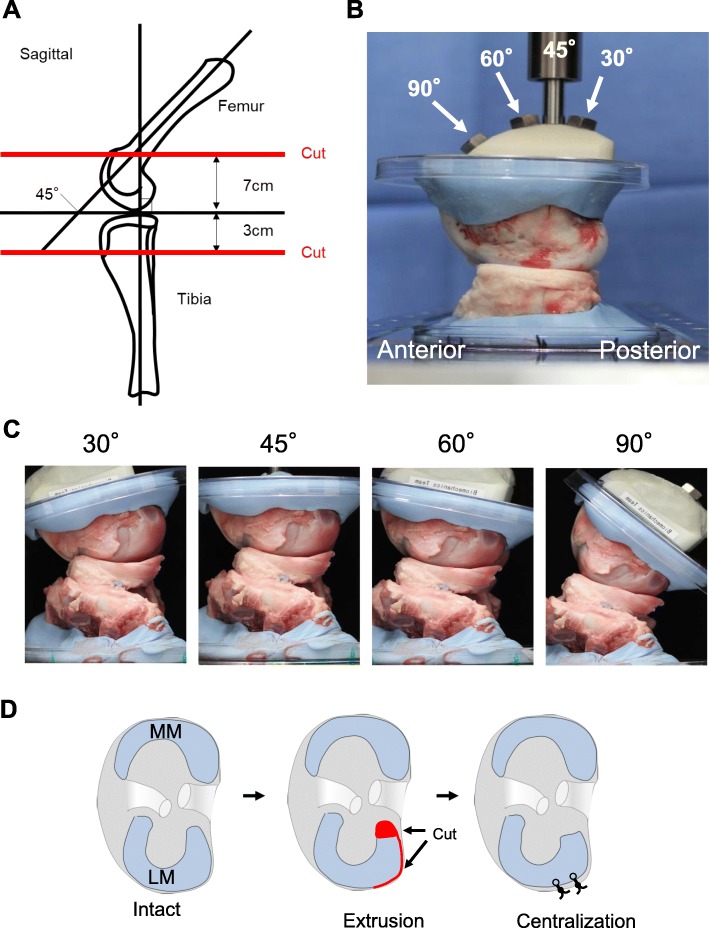
Experimental settings. **a** Schematic diagram for cut line of the femur and tibia. **b** Angle changing device set at 45°. **c** Intact porcine knees viewed laterally, set at 30°, 45°, 60°,and 90° flexion. **d** Scheme for extrusion and centralization. LM, lateral meniscus; MM, medial meniscus

After the muscles were removed, we cut the tibia bone horizontally at 3 cm distal to the joint and the femoral bone obliquely at 45° at 7 cm proximal from the joint. Then, we fixed the femur bone and tibia bone with a tester using polymethyl methacrylate (Fig. 1a). We cut the lateral collateral ligament (LCL) and inserted the sensor seat. We preserved the medial collateral ligament, anterior cruciate ligament (ACL), and posterior cruciate ligament (PCL). The angle-changing device was placed between the knee and a universal testing machine (Fig. 1b), so that the knee flexion angles could be set at 30°, 45°, 60°, and 90° (Fig. 1c).

The mechanical setup was as follows: 1) Intact; 2) Extrusion—we removed a 1 cm width of the posterior root of the LM to preclude anatomical repair, cut the posterior capsule horizontally from the posterior root attachment site of the posterior root, and created meniscal extrusion (Fig. 1d); and 3) Centralization—we inserted the first 1.4 mm soft anchor (JuggerKnot, Zimmer Biomet, Warsaw, IN, USA) into the lateral tibial plateau 1 cm anterior to the popliteal hiatus, the second 1.4 mm soft anchor into the lateral tibial plateau 1 cm anterior to the first anchor, and reduced the extruded meniscus to its original position (Fig. 1d). We passed the sutures through the border between the meniscus and the remaining capsule attached to the meniscus and secured to the tibia using mattress sutures. For all angles, an axial compressive force of 200 N was applied in each setting [15, 18].

### Deviation distance of the lateral meniscus

**Fig. 2:**
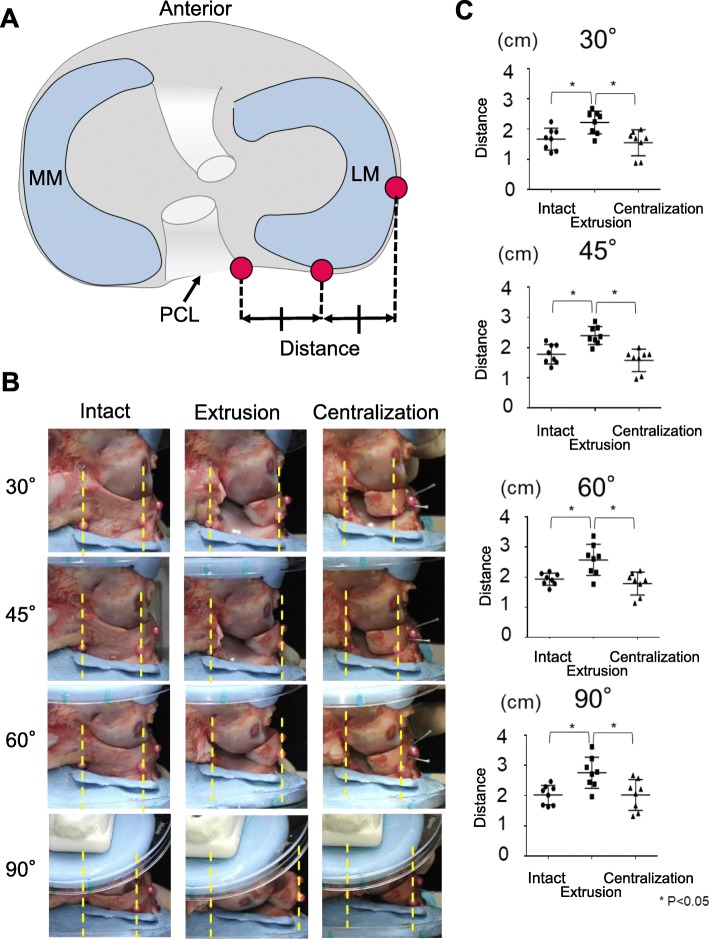
LM deviation distance with an axial compressive force of 200 N. **a** Marker locations. PCL, posterior cruciate ligament. **b** Knee viewed posteriorly. **c** Quantitative analysis of inter-line distances. Each bar represents the average with 95% CI (*n* = 8). (*P < 0.05)

Three spherical red plastic markers (3 mm diameter) were attached: the posterior marker at the center of the tibial attachment of the PCL; the lateral marker at the lateral edge of the LM in the posterior view; and the posterolateral marker at the point where the middle line of the other two points intersects the outer edge of the meniscus in the posterior view (Fig. 2a). We placed the markers anterior to the resection. After application of an axial compressive force of 200 N, the LM was photographed in the posterior view and the distance between the posterior marker line and the posterolateral marker line measured to evaluate the meniscal extrusion (Fig. 2a).

### Contact area and force measurements

We used a pressure mapping sensor (Tekscan, Inc. South Boston, MA, USA) to quantify the distribution of load-bearing force on the lateral compartment. Tekscan enabled electronic scanning to measure the real-time force and contact area. We placed the sensor on the femoral side of the lateral meniscus and recorded the load distribution, as well as contact area, maximum contact pressure, and average contact pressure. We analyzed the data with MATLAB® (MathWorks, MA, USA).

### Statistics

We used the Friedman one-way non-parametric test and Dunn’s test as post hoc tests using Prism 6 software (GraphPad Inc., La Jolla, CA, USA). *P* values of less than 0.05 were considered statistically significant. All data were shown as means with 95% confidence intervals (CI).

## Results

The distance between the two markers significantly increased after extrusion at all flexion angles (Fig. 2b, c; Supplementary Table 1). Conversely, it significantly decreased after centralization at each flexion angle. In all settings, the distance between the two markers increased with the knee flexion angle, although there were no significant differences among the distances measured for each angle (Supplementary Table 1).

**Fig. 3:**
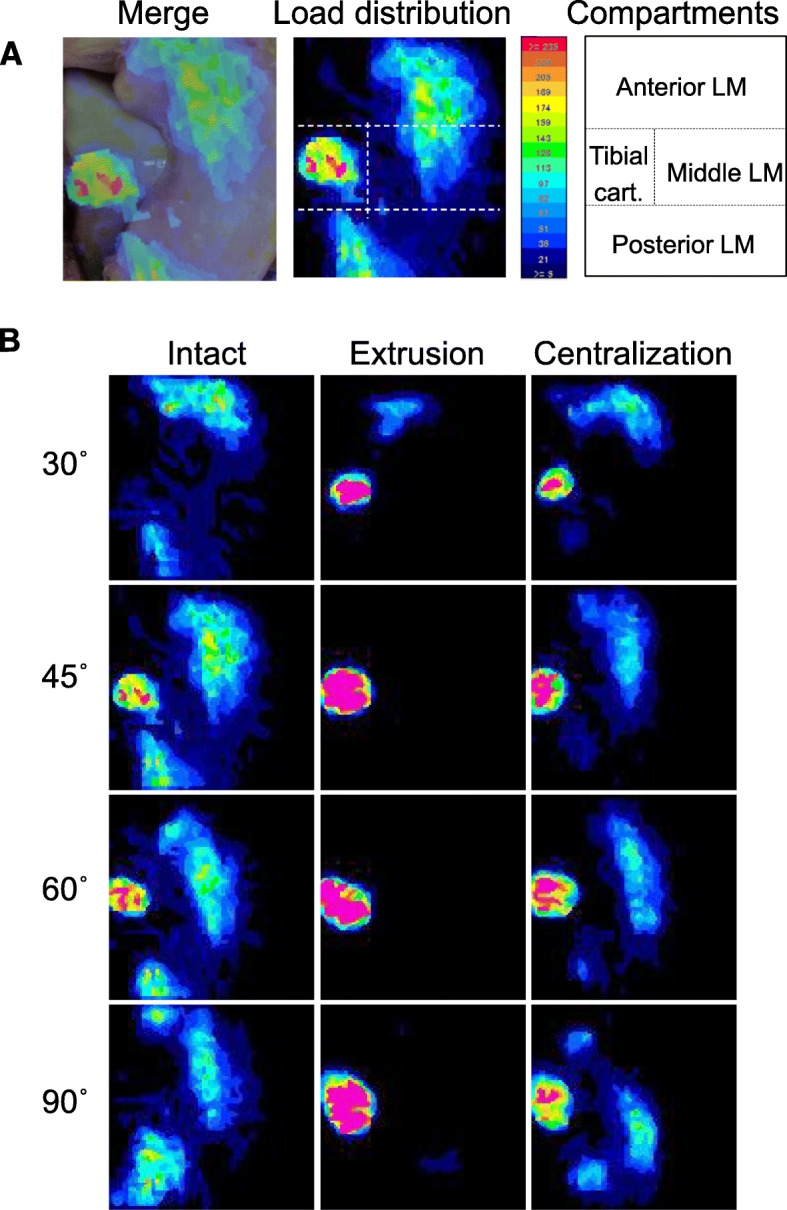
Load distribution analyzed with a pressure mapping sensor system. **a** Tibial cartilage with LM; superposed image of load distribution and macro picture, lateral tibial surface divided into four compartments. **b** Representative load distribution, axial compressive force 200 N

For load distribution analyses, the lateral compartment was divided into the anterior LM, the middle LM, the posterior LM, and the tibial cartilage areas (Fig. 3a). For each angle, according to the representative images (Fig. 3b), the load was concentrated on the tibial cartilage after extrusion and redistributed to the anterior and middle LM after centralization.

**Fig. 4:**
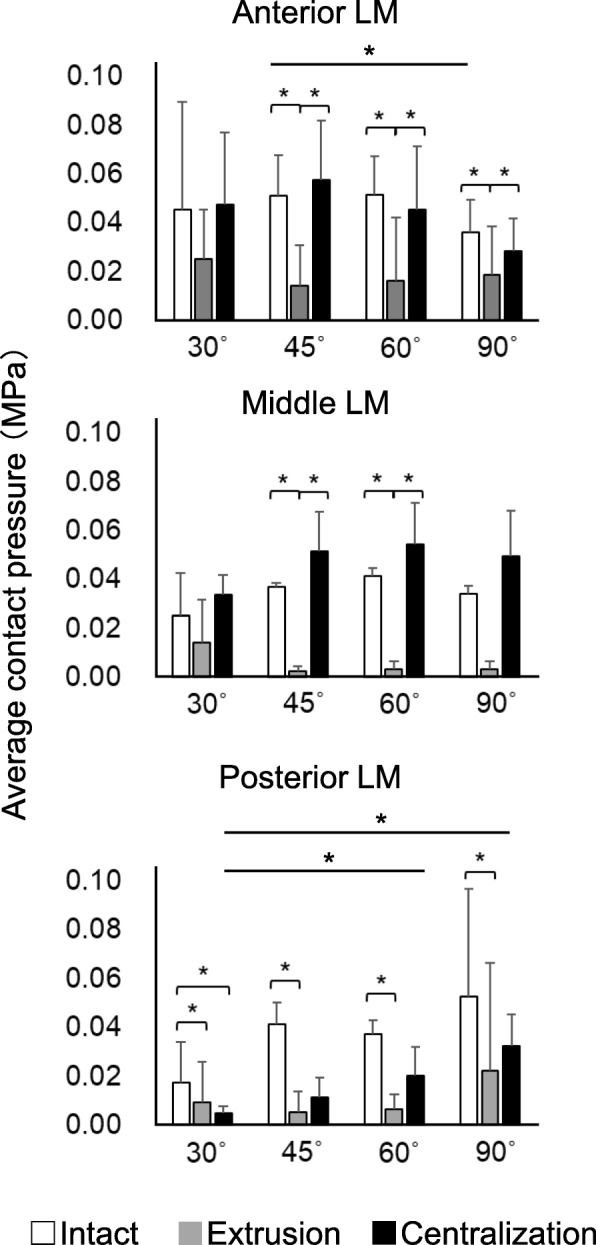
Quantitative analyses of average contact pressure on the anterior, middle, and posterior LM. The average values with 95% CI are shown (*n* = 8). (*P < 0.05)

**Fig. 5:**
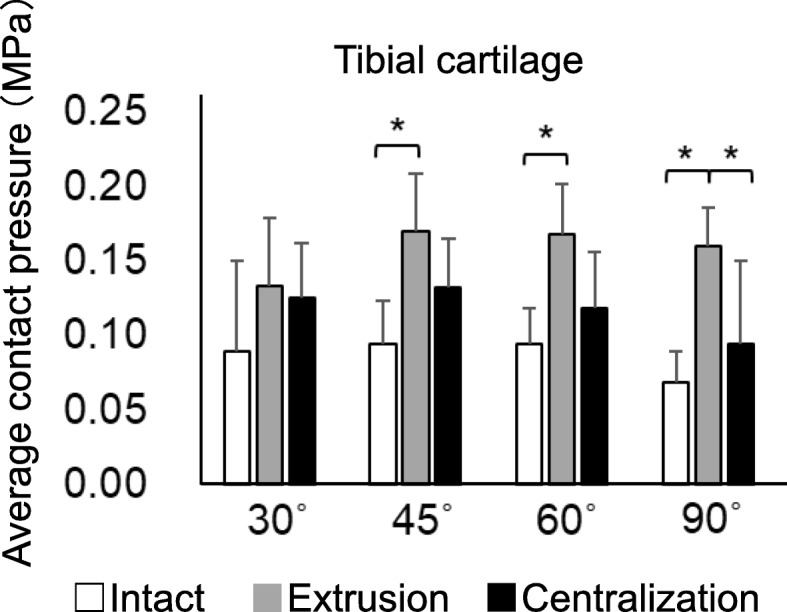
Quantitative analyses of average contact pressure on the lateral tibial cartilage. The average values with 95% CI are shown (*n* = 8). (*P < 0.05)

The average contact pressure in the anterior and middle LM decreased significantly after extrusion and increased after centralization. Similar results were obtained for each flexion angle, except in the anterior LM at 30° and the middle LM at both 30° and 90° (Fig. 4, Supplementary Table 2). On the other hand, while extrusion significantly decreased the average contact pressure in the posterior LM, centralization did not fully restore it. Similar results were obtained for each flexion angle. In the anterior LM, the average contact pressure at 45° significantly decreased at 90° in the intact setting; conversely, in the posterior LM, the average contact pressure at 30° significantly increased at 60° and 90° in the centralization setting. In the tibial cartilage, extrusion significantly increased the average contact pressure at 45°, 60°, and 90°, whereas centralization significantly decreased it at 90° (Fig. 5, Supplementary Table 3).

**Fig. 6:**
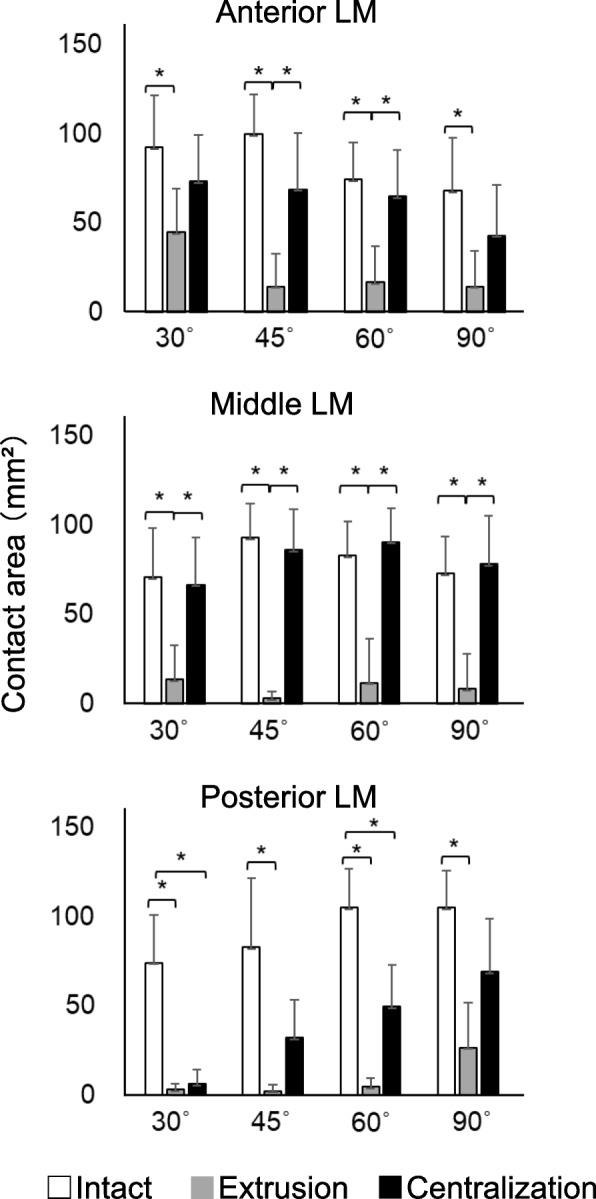
Quantitative analyses of contact area in the anterior, middle, and posterior LM. The average values with 95% CI are shown (*n* = 8). (*P < 0.05)

The contact area significantly decreased after extrusion at each flexion angle in the anterior, middle, and posterior LM (Fig. 6, Supplementary Table 4). Contrarily, it significantly increased after centralization at each flexion angle in the anterior and middle LM, whereas centralization did not fully recover the contact area in the posterior LM. In all settings, the contact area in the anterior LM appeared to decrease with the knee flexion angle, although there were no significant differences among the areas measured for each angle (Supplementary Table 4). Also, in all settings, the contact area in the posterior LM appeared to increase with knee flexion angle, although there were no significant differences.

## Discussion

In this study, the biomechanics of the extruded and centralized LM were analyzed in porcine knee joints at different flexion angles. In the anterior and middle LM, both the contact pressure and area decreased in extrusion, increasing close to the intact status after the centralization procedure. In this model, the effectiveness of centralization to restore the lost function of the meniscus has been demonstrated in the anterior and middle LM.

We set knee flexion angles at 30°, 45°, 60°, and 90°. Since the most extended position is approximately at 30° and the most flexed position is approximately at 90° in a pig knee joint, we first set the flexion angles every 30° between 30° and 90°. We also set 45° because we set that angle in our previous report [15]. Biomechanical analysis was therefore performed at 30°, 45°, 60°, and 90°.

We applied 200 N as an axial compressive force at each setting. The body weight of the pigs which knees we used was approximately 80–100 kg. The pig’s center of gravity is near the forelegs and the load on the hind legs is lower than on the forelegs. When standing on a quadruped, the load on single hind leg of an 80–100 kg pig is approximately 160–200 N [19]. Two hundred N might be too small as an axial compressive force for biomechanical studies. However, in this study, 200 N was sufficient to examine the effects of meniscal extrusion and the effects of centralization. In our previous report, 200 N was the force applied in a similar setting [15]. Furthermore, reported that, in their model, which used similar porcine knees, the in situ force of the LM with a complete radial tear significantly decreased even under an axial load of 150 N [17]. Therefore, the axial compressive force applied in this study would be large enough to yield clinically significant findings.

Although the deviation distance of the LM, which increased in extrusion, was restored to the intact status in centralization at all angles, the contact pressure and area, decreased in extrusion, were not fully restored in the posterior LM, even after centralization. This was possibly because a 1 cm width of the posterior root deficiency was left untreated. These results suggest that hoop function should also be reconstructed, if possible, in order to fully restore the load distribution function of the posterior LM. Even so, centralization decreased the contact pressure in the tibial cartilage, and this effect became more obvious as the flexion angle became larger.

The distances between the two markers increased with knee flexion angle in each setting, although no significances were found. This can be explained from the results of the current study; the load distribution moved posteriorly as the flexion angle increased. A previous magnetic resonance imaging (MRI) study also supports our results, showing that the lateral femoral condyle and LM consistently displayed a marked posterior translation [20].

To our knowledge, previous reports of biomechanical analysis for the centralization of the extruded meniscus are limited. Nakamura et al. used the centralization procedure in an ACL-reconstructed porcine knee with an irreparable lateral meniscus defect to evaluate the effects of knee biomechanics; they reported that using arthroscopic centralization for the capsular support of the middle segment of the lateral meniscus improved the residual rotational laxity of the ACL-reconstructed knee, which had lateral meniscus dysfunction due to massive meniscal defect [17]. Daney et al., in the only report other than ours [15], measured meniscal extrusion and tibiofemoral contact mechanics at the medial compartment in human cadaveric knees [16]. The anatomic transtibial pull-out root repair and the anatomic transtibial pull-out root repair with centralization suture techniques best restored the contact mechanics of the knee and meniscal extrusion when compared with root tear and nonanatomic repair states. However, the degree of extrusion increased as the knee was flexed to 90°. Their study differs from ours in terms of using human knees, examining the inner compartment, and performing the centralization with pullout techniques; both studies, however, showed the effectiveness of centralization.

We previously reported the effect of centralization in a porcine model [15]. The methods used in the two studies were similar in that the experimental settings were the same. The difference between the two studies was that, in the current study, biomechanical analysis was performed at 30°, 60°, and 90° as well as at 45°. Similar results were obtained at 45° and the new findings were revealed at 30°, 60°, and 90° in knee flexion. Although significant differences of contact area and contact pressure at different angles were not detected, the following trends were observed: Contact area and contact pressure in the anterior and middle LM reached their maxima at 30°, 45°, and 60°, while those at the posterior LM reached theirs at 90°.

For limitations, we cut the lateral collateral ligament to insert a sensor from the lateral side; this raised a concern that instability caused by LCL deficiency could have affected the results. We also inserted a pressure mapping sensor between the femoral cartilage and the LM, rather than between the LM and the tibial cartilage, which would have impaired the load-distribution measurements for the entire tibial cartilage. However, the knee joint was stabilized and the loading force applied in the vertical direction; the comparison of evaluated values under intact, extrusion, and centralization settings at each flexion angle will therefore provide important information.

## Conclusions

The centralization procedure could reduce extrusion of the LM and restore the load distribution function of the anterior-middle LM in a porcine model. However, the procedure itself could not restore hoop function in cases with a defect of the posterior LM.

## Supplementary information

**Additional file 1: Supplementary Table S1.** Distance between two markers at 200 N loading. **Supplementary Table S2.** Average contact pressure for anterior, middle, and posterior LM. **Supplementary Table S3.** Average contact pressure for the lateral tibial cartilage. **Supplementary Table S4.** Contact area for the anterior, middle, and posterior LM.

## Data Availability

All data generated or analysed during this study are included in this published article.

## Abbreviations

OA: Osteoarthritis
LM: Lateral meniscus
ACL: Anterior cruciate ligament
LCL: Lateral collateral ligament
PCL: Posterior cruciate ligament
CI: Confidence intervals
MRI: Magnetic resonance imaging
