# Supplementary material for: The effect of a centralization procedure for extruded lateral meniscus on load distribution in porcine knee joints at different flexion angles
Source: BMC Musculoskelet Disord. 2020 Apr 3;21:205. doi: 10.1186/s12891-020-03197-2 (PMC7126455; doi:10.1186/s12891-020-03197-2)
Supplement: Supplementary file 1 — Additional file 1: Supplementary Table S1. Distance between two markers at 200 N loading. Supplementary Table S2. Average contact pressure for anterior, middle, and posterior LM. Supplementary Table S3. Average contact pressure for the lateral tibial cartilage. Supplementary Table S4. Contact area for the anterior, middle, and posterior LM. [file 12891_2020_3197_MOESM1_ESM.docx]

**Supplementary Table 1. Distance between two markers at 200 N loading.**

|  |  | **Distance (cm)** |
| --- | --- | --- |
| **30°** | **Intact** | 1.67  (1.42~1.92) |
|  | **Extrusion** | 2.22^a^  (1.98~2.48) |
|  | **Centralization** | 1.55^b^  (1.25~1.85) |
| **45°** | **Intact** | 2.0  (1.56~2.0) |
|  | **Extrusion** | 2.40^a^  (2.19~2.60) |
|  | **Centralization** | 1.58^b^  (1.32~1.84) |
| **60°** | **Intact** | 1.93  (1.80~2.07) |
|  | **Extrusion** | 2.58^a^  (2.22~2.93) |
|  | **Centralization** | 1.79^b^  (1.53~2.06) |
| **90°** | **Intact** | 2.02  (1.80~2.25) |
|  | **Extrusion** | 2.76^a^  (2.40~3.11) |
|  | **Centralization** | 2.02^b^  (1.67~2.38)  *^a^  (,)  *^a^  (,) |

Average values with 95% CI for 8 samples are shown.

^a^ p< 0.05 between the Intact group and the Extrusion group.

^b^ p < 0.05 between the Extrusion group and the Centralization group.

**Supplementary Table 2. Average contact pressure for anterior, middle, and posterior LM.**

|  |  | **Average contact pressure (MPa)** | | |
| --- | --- | --- | --- | --- |
| **30°** |  | **Anterior** | **Middle** | **Posterior** |
|  | **Intact** | 0.046  (0.015~0.076) | 0.025  (0.015~0.035) | 0.017^d^  (0.012~0.023) |
|  | **Extrusion** | 0.025  (0.011~0.039) | 0.014  (0.002~0.026)  ,) | 0.009^a^  (-0.002~0.021) |
|  | **Centralization** | 0.047  (0.027~0.068) | 0.034  (0.023~0.044) | 0.005^cd^  (0.003~0.007) |
| **45°** |  | **Anterior** | **Middle** | **Posterior** |
|  | **Intact** | 0.051^d^  (0.04~0.076) | 0.037  (0.023~0.051) | 0.028  (0.017~0.039) |
|  | **Extrusion** | 0.014^a^  (0.003~0.026) | 0.003^ab^  (0.001~0.004) | 0.005^a^  (-0.001~0.011) |
|  | **Centralization** | 0.057^b^  (0.041~0.074) | 0.052^b^  (0.036~0.067) | 0.011  (0.006~0.017) |
| **60°** |  | **Anterior** | **Middle** | **Posterior** |
|  | **Intact** | 0.052  (0.04~0.062) | 0.042  (0.03~0.053) | 0.037  (0.025~0.049) |
|  | **Extrusion** | 0.016^a^  (-0.002~0.034) | 0.004^a^  (0.001~0.006) | 0.007^a^  (-0.008~0.011) |
|  | **Centralization** | 0.046^b^  (0.028~0.063) | 0.054^b^  (0.038~0.071) | 0.02  (0.012~0.028) |
| **90°** |  | **Anterior** | **Middle** | **Posterior** |
|  | **Intact** | 0.036^d^  (0.027~0.046) | 0.034  (0.023~0.045) | 0.053^d^  (0.04~0.066) |
|  | **Extrusion** | 0.019^a^  (0.05~0.032) | 0.003  (0.001~0.006) | 0.022^a^  (-0.008~0.053) |
|  | **Centralization** | 0.028^b^  (0.019~0.038) | 0.049  (0.032~0.067) | 0.032^d^  (0.023~0.041) |

Average values with 95% CI for 8 samples are shown.

^a^ p< 0.05 between the Intact group and the Extrusion group; ^b^ p < 0.05 between the Extrusion group and the Centralization group; ^c^ p < 0.05 between the Intact group and the Centralization group; and ^d^ p < 0.05 between the 30° or 45°group and the 90°group.

**Supplementary Table 3. Average contact pressure for the lateral tibial cartilage.**

|  |  | **Average contact pressure (MPa)** |
| --- | --- | --- |
| **30°** | **Intact** | 0.09  (0.048~0.132) |
|  | **Extrusion** | 0.133  (0.101~0.165) |
|  | **Centralization** | 0.125  (0.099~0.151) |
| **45°** | **Intact** | 0.095  (0.075~0.114) |
|  | **Extrusion** | 0.17^a^  (0.143~0.197) |
|  | **Centralization** | 0.132  (0.109~0.155) |
| **60°** | **Intact** | 0.095  (0.079~0.111) |
|  | **Extrusion** | 0.168^a^  (0.144~0.192) |
|  | **Centralization** | 0.118  (0.092~0.144) |
| **90°** | **Intact** | 0.068  (0.054~0.083) |
|  | **Extrusion** | 0.161^a^  (0.143~0.178) |
|  | **Centralization** | 0.095^b^  (0.056~0.133) |

Average values with 95% CI for 8 samples are shown.

^a^ p< 0.05 between the Intact group and the Extrusion group.

^b^ p < 0.05 between the Extrusion group and the Centralization group.

**Supplementary Table 4. Contact area for the anterior, middle, and posterior LM.**

|  |  | **Contact area (mm^2^)** | | |
| --- | --- | --- | --- | --- |
| **30°** |  | **Anterior** | **Middle** | **Posterior** |
|  | **Intact** | 92.5  (72.6~112.5) | 70.9  (51.8~90.0) | 74.3  (55.9~92.6) |
|  | **Extrusion** | 45.0^a^  (28.0~61.9) | 13.8^a^  (0.5~27.0) | 3.3^a^  (0.9~5.6) |
|  | **Centralization** | 73.3  (55.2~91.3) | 66.9^b^  (48.8~84.9) | 6.6^c^  (1.3~11.9) |
| **45°** |  | **Anterior** | **Middle** | **Posterior** |
|  | **Intact** | 100  (84.7~115.3) | 93.1  (80.2~106.0) | 96.8  (69.8~123.7) |
|  | **Extrusion** | 14.6  (2.0~27.2) | 3.1^a^  (0.7~5.9) | 2.5^a^  (0~5.0) |
|  | **Centralization** | 69.0  (47.1~90.9) | 86.0^b^  (70.0~101.9) | 32.5  (17.9~47.1) |
| **60°** |  | **Anterior** | **Middle** | **Posterior** |
|  | **Intact** | 74.5  (60.2~88.7) | 82.8  (69.3~96.2) | 105.3  (90.4~120.1) |
|  | **Extrusion** | 17.0^a^  (3.1~30.9) | 12.0^a^  (-4.9~28.9) | 4.9^a^  (1.5~8.3) |
|  | **Centralization** | 65.0^b^  (47.1~82.9) | 90.6^b^  (77.7~101.9) | 49.6^c^  (17.9~47.1) |
| **90°** |  | **Anterior** | **Middle** | **Posterior** |
|  | **Intact** | 68.1  (47.5~88.8) | 72.9  (58.4~87.3) | 105.3  (91.0~119.5) |
|  | **Extrusion** | 14.6^a^  (0.9~30.9) | 8.6^a^  (-5.0~22.2) | 26.8^a^  (9.3~44.1) |
|  | **Centralization** | 43.1  (23.4~62.9) | 78.0^b^  (59.2~96.8) | 69.1  (48.7~89.6) |

Average values with 95% CI for 8 samples are shown.

^a^ p< 0.05 between the Intact group and the Extrusion group

^b^ p < 0.05 between the Extrusion group and the Centralization group;

^c^ p < 0.05 between the Intact group and the Centralization group.
